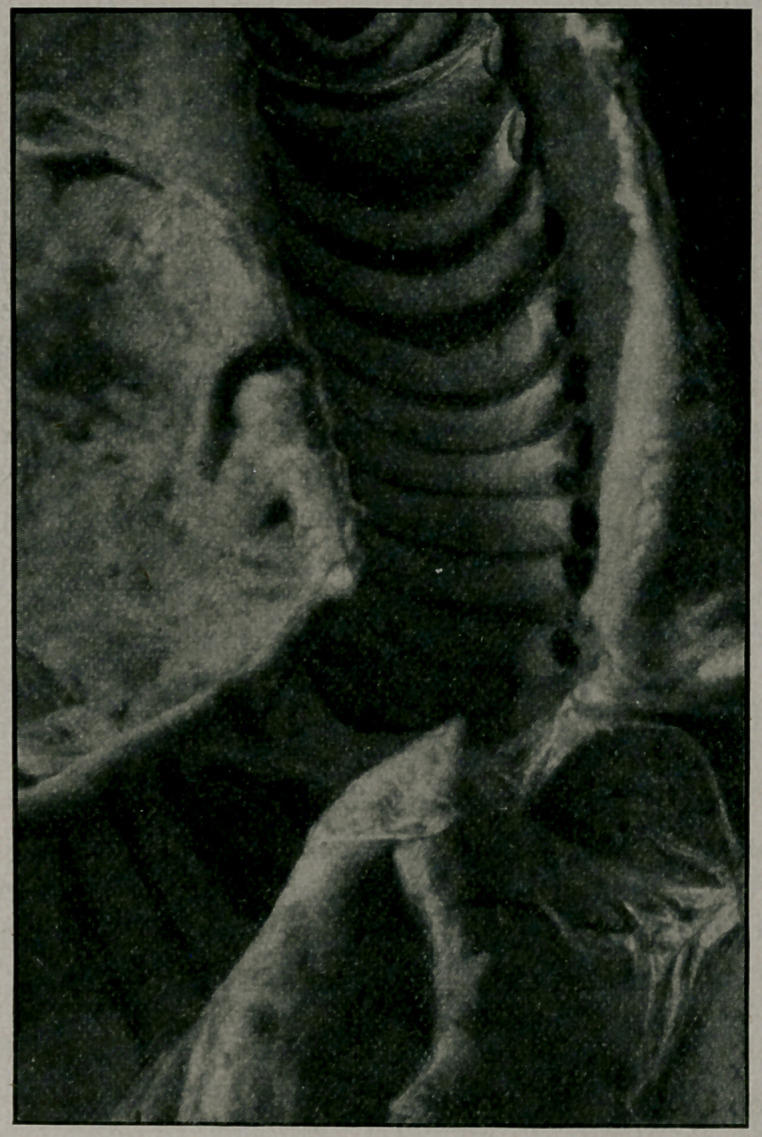# Syphilitic Tracheal Stenosis

**Published:** 1913-02

**Authors:** 


Syphilitic Tracheal Stenosis.—Dr. E. H. Reede of Washington, D. C., *Wash. Med. Annals,* November, 1912, reports a fatal case in a woman aged 32 and weighing 200 pounds. A number of suffocative attacks occurred in the fourteen months ending in her death, July 12, 1912. A cicatrix was found at the bifurcation of the trachea, the lumen being only 2 m. m. in diameter; three other cicatrices were found in the principal bronchi. The diagnosis of syphilis rested solely on these findings. A review of literature follows and the article is a most valuable monograph. Through the courtesy of the author and the editor, we are allowed to reproduce the accompanying illustration.

## Figures and Tables

**Figure f1:**